# Rutile Without Substrate Limitations: Top‐Interface‐Driven Crystallization of TiO_2_

**DOI:** 10.1002/adma.73040

**Published:** 2026-04-09

**Authors:** Jihoon Jeon, Jongseo Kim, Seungwan Ye, Seong Keun Kim

**Affiliations:** ^1^ Electronic and Hybrid Materials Research Center Korea Institute of Science and Technology Seoul Republic of Korea; ^2^ KU‐KIST Graduate School of Converging Science and Technology Korea University Seoul Republic of Korea

**Keywords:** atomic layer deposition, DRAM capacitors, high‐k dielectrics, rutile, substrate‐independent phase stabilization, top‐interface‐driven crystallization

## Abstract

Controlling the polymorphic phases within the thermal budget of atomic layer deposition (ALD) is essential for integrating high‐k dielectrics into dynamic random‐access memory (DRAM) capacitors. Rutile TiO_2_ offers a dielectric constant significantly higher than that of tetragonal ZrO_2_ and anatase TiO_2_. However, its application on industry‐standard TiN electrodes is impeded by the lack of rutile‐compatible lattice matching. A top‐interface‐driven stabilization strategy is demonstrated, where a structurally compatible RuO_2_ upper layer stabilizes rutile TiO_2_ at 400°C regardless of the crystallinity of the underlying ZrO_2_/TiN stack. Thickness‐dependent phase maps reveal an interfacial‐energy‐driven anatase‐to‐rutile transition for thin amorphous TiO_2_ layers, enabling rutile formation even on amorphous ZrO_2_. The resulting TiO_2_/ZrO_2_/TiN capacitors exhibit a dielectric constant of approximately 80 and a reduced equivalent oxide thickness, comparable to that of ZrO_2_‐based stacks. A methanol‐assisted reduction‐etching process allows selective removal of RuO_2_ by O_3_ with minimal TiN oxidation. This top‐interface engineering concept offers a substrate‐agnostic approach to rutile TiO_2_ that is compatible with DRAM process windows and can be extended to other polymorphic oxides.

## Graphical abstract

A RuO_2_ upper layer acts as a removable crystallization template that triggers rutile TiO_2_ formation from the top interface, independent of the underlying substrate. This top‐interface‐driven crystallization mechanism enables rutile growth even on amorphous ZrO_2_/TiN, offering a substrate‐agnostic route to high‐k dielectrics compatible with DRAM process conditions.

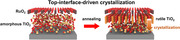

## 1 Introduction

Polymorphs are phases with identical chemical formulas but different crystal structures, and certain forms are challenging to synthesize using conventional methods. The stabilization of these hard‐to‐form phases has garnered significant attention as a key strategy for enhancing material properties, especially in thin film synthesis for advanced electronic devices [1, 2, 3]. However, the conditions necessary for producing these films often conflict with those employed in standard film growth procedures or with the limited thermal budgets available during device manufacturing [4, 5, 6, 7]. This challenge is particularly pronounced in atomic layer deposition (ALD), a widely utilized technique in nanoelectronics, as the process temperature must stay below the decomposition limit of the precursor.

The most commonly employed strategy for stabilizing hard‐to‐form polymorphs within the restricted thermal budget of ALD involves using a substrate with lattice matching to the desired structure [8, 9, 10, 11, 12]. Utilizing substrates that share the same crystal structure [8, 9], or possess crystallographic planes compatible with those of the film [10, 13], provides an effective means to stabilize challenging phases. Despite its significant potential, this method has limited applicability as it depends on a specific substrate and is restricted to certain film‐substrate material systems. Hence, substrate‐independent approaches are essential for expanding the range of attainable hard‐to‐form phases.

Rutile TiO_2_ is a representative hard‐to‐form oxide, for which substrate‐independent ALD growth is essential. Owing to its exceptionally high dielectric constant (80 along the a‐axis and 170 along the c‐axis) [14, 15], which far exceeds those of both the currently adopted tetragonal ZrO_2_ (∼40) and the stable anatase TiO_2_ phase (∼40), rutile TiO_2_ is regarded as a promising dielectric layer for next‐generation dynamic random‐access memory (DRAM) capacitors [16, 17, 18, 19]. To stabilize the rutile phase within the limited thermal budget (<< 500°C) allowed for fabrication of DRAMs, conductive oxides such as RuO_2_ [15, 20], IrO_2_ [21], MoO_2_ [12, 22, 23], and SnO_2_ [24] that have structural compatibility with rutile TiO_2_ have been investigated as bottom electrodes. However, these oxide layers are typically unstable, difficult to synthesize, and incompatible with conventional fabrication processes. Recently, a sacrificial layer strategy enabling the formation of rutile TiO_2_ without substrate limitations has been reported [25], suggesting a promising direction for substrate‐independent stabilization. Still, the key challenge for applying rutile TiO_2_ dielectrics in DRAM capacitors is not only achieving substrate‐independent growth but also achieving it on the industry‐standard TiN electrode, which is not structurally compatible with rutile. This is essential for the practical integration of rutile TiO_2_ into commercial DRAM devices.

In this study, we present a new approach to synthesizing rutile TiO_2_ at reduced temperatures by utilizing a structurally compatible upper layer instead of depending on lattice matching with the underlying layer. This method, driven by the top interface, allows for the stabilization of rutile TiO_2_ while retaining the conventional TiN bottom electrodes, offering a feasible route for incorporating rutile TiO_2_ into device structures. Furthermore, by separating the formation of metastable phases from substrate constraints, this strategy is not limited to specific materials and could serve as a broadly applicable method for controlling metastable phases within ALD processing windows.

## 2 Results and Discussion

**FIGURE 1:**
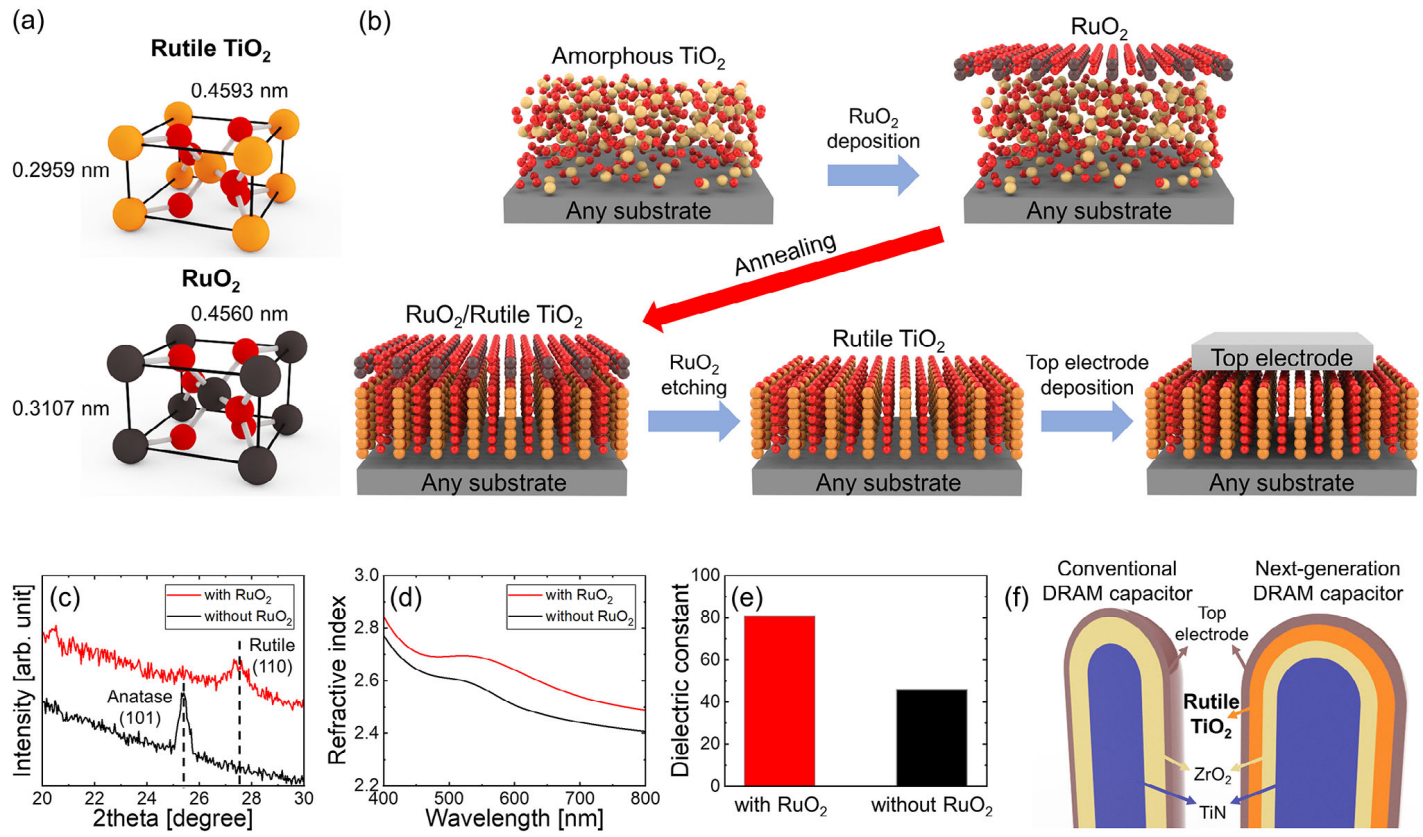
(a) Unit‐cell structures of rutile TiO_2_ and RuO_2_. (b) Schematic of the rutile formation process using the top‐interface‐driven stabilization strategy. (c) GIXRD patterns, (d) refractive index, and (e) dielectric constant of TiO_2_ films grown on ultrathin ZrO_2_/TiN stack, with and without the top‐interface‐driven approach. (f) Conceptual DRAM capacitor design integrating rutile TiO_2_ on ZrO_2_/TiN to lower EOT while retaining leakage suppression.

In this study, RuO_2_ was utilized as a structurally compatible upper layer. As depicted in Figure 1a, RuO_2_ shares the same crystal structure as rutile TiO_2_. Additionally, the lattice constants (a = 0.4499 nm and c = 0.3107 nm) closely resembled those of rutile TiO_2_ (a = 0.4593 nm and c = 0.2959 nm). The lattice mismatches were minimal, approximately 2.1% along the a‐axis and −4.8% along the c‐axis. Owing to its structural compatibility, RuO_2_ has been widely employed as a bottom electrode to stabilize the rutile phase of TiO_2_ films through ALD even at low temperatures (Figure S1) [15, 20, 26, 27, 28, 29].

A strategy using RuO_2_ as the upper layer to stabilize the underlying TiO_2_ layer in the rutile phase is depicted in Figure 1(b). First, an amorphous TiO_2_ film was deposited through ALD on a non‐lattice‐matched bottom layer, including the amorphous layers. Subsequently, a RuO_2_ upper layer was applied and then subjected to annealing. The as‐grown RuO_2_ upper layer was already crystallized into the rutile phase prior to annealing (Figure S2). It is anticipated that during annealing, the RuO_2_/TiO_2_ top‐interface will promote crystallization into the rutile phase. Following annealing, the RuO_2_ layer was selectively eliminated, and an appropriate conducting layer was established as the top electrode for capacitor production.

The top‐interface‐driven strategy not only overcomes substrate limitations for achieving rutile TiO_2_ formation but also allows flexibility in selecting top electrode materials. While RuO_2_ is conductive enough to function as an electrode, its chemical instability in a reducing atmosphere, like during forming gas annealing for trap passivation in transistors, can cause electrode degradation [30]. In contrast, RuO_2_ can be easily etched by oxidation into volatile RuO_4_, distinguishing it from other rutile‐compatible conducting oxides such as doped SnO_2_ and MoO_2_, which lack this etching selectivity with TiO_2_. Considering the structural compatibility and process versatility enabled by selective removal, RuO_2_ was selected as the upper‐layer material to induce rutile formation in this study.

The structural engineering of TiO_2_ films using this strategy is highly effective. The grazing‐incident X‐ray diffraction (GIXRD) patterns in Figure 1c demonstrate that the TiO_2_ film directly grown on the ultrathin ZrO_2_/TiN crystallized into the anatase phase, while the addition of a RuO_2_ upper layer stabilized the rutile phase. The formation of rutile TiO_2_ is further supported by the higher refractive index observed compared to the film without the upper layer (Figure 1d), consistent with the inherently higher refractive index of rutile compared to anatase [10, 14]. Capacitors fabricated with TiN bottom electrodes using this RuO_2_‐top‐interface‐driven approach exhibit a significant increase in the dielectric constant of the TiO_2_ films (Figure 1e). The alignment of structural, optical, and electrical data confirms that the top‐interface‐driven stabilization method enables substrate‐independent creation of rutile TiO_2_.

Currently, DRAM capacitors utilize ZrO_2_‐based dielectrics along with TiN electrodes. To enhance the scaling of the equivalent oxide thickness (EOT) while minimizing leakage currents, a capacitor design that substitutes part of the ZrO_2_ layer with rutile TiO_2_, as illustrated in Figure 1f, emerges as a promising option for next‐generation DRAMs. In this structure, the rutile TiO_2_ layer effectively reduces the EOT, while the remaining ZrO_2_ layer in contact with TiN maintains the Schottky barrier and suppresses leakage currents. As a result, the proposed top‐interface‐driven approach holds the potential to facilitate the production of such capacitor structures using process conditions compatible with conventional DRAM manufacturing.

**FIGURE 2:**
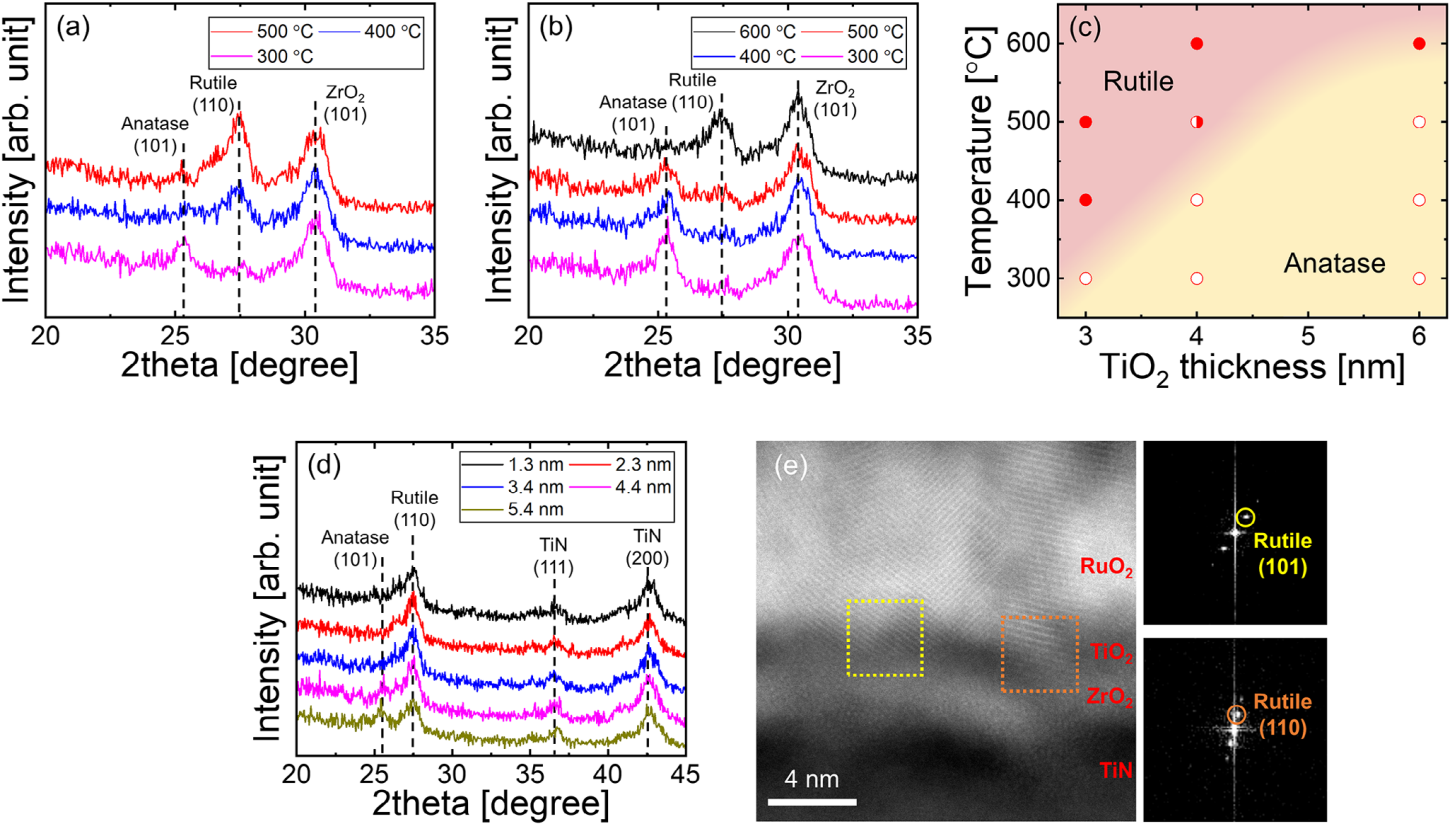
GIXRD patterns of TiO_2_ films grown using the top‐interface‐driven strategy with amorphous TiO_2_ seed layers of (a) 3 nm and (b) 6 nm on a 10 nm‐thick crystalline ZrO_2_ layer/SiO_2_/Si. (c) Thickness–annealing temperature phase map summarizing the anatase–rutile transition of the amorphous TiO_2_ layer using this strategy. (d) GIXRD patterns of TiO_2_ films grown from 1.3–5.4 nm‐thick TiO_2_ seed layers on 1 nm‐thick amorphous ZrO_2_/TiN stacks. (e) Cross‐sectional TEM image of RuO_2_/TiO_2_/ZrO_2_/TiN stacks with amorphous ZrO_2_, with insets showing fast Fourier transform images of the boxed region confirming rutile formation.

To investigate the crystallization behavior of TiO_2_ films with the proposed structure, amorphous TiO_2_ films were deposited on ZrO_2_ surfaces, and their crystalline phases were examined with respect to the annealing temperature. Figure 2a,b shows the GIXRD patterns of TiO_2_ films grown using (a) 3 nm‐ and (b) 6 nm‐thick TiO_2_ seed layers formed on 10 nm‐thick crystalline ZrO_2_, respectively. The 6 nm‐thick TiO_2_ seed layer was identified as amorphous (Figure S3). For easy identification of the phase, an additional 25 nm‐thick TiO_2_ layer was deposited after the crystallization annealing and subsequent etching of RuO_2_. For both the TiO_2_ films using 3 and 6 nm‐thick seed layers, a phase transition from anatase to rutile was observed with increasing annealing temperature. However, rutile formation occurred at a lower temperature of 400°C for the 3 nm‐thick seed layer, whereas the transition temperature increased to 600°C for the 6 nm‐thick seed layer. By integrating the results for various seed layer thicknesses (Figure S4), a phase map showing the anatase‐rutile transition concerning the amorphous TiO_2_ thickness and annealing temperature is illustrated in Figure 2c. This indicates that a thicker amorphous TiO_2_ layer requires higher thermal energy for stabilization into the rutile phase.

This observation can be understood as a result of the competition between the interfacial energy and the bulk free energy of the TiO_2_ seed layer during crystallization annealing. For thinner TiO_2_ layers, the interfacial energy has a greater impact than the bulk free energy, while the significance of the bulk free energy increases with layer thickness. The structural compatibility between the RuO_2_ upper layer and the rutile TiO_2_ significantly reduces the interfacial energy for rutile nucleation. Therefore, rutile formation is more favorable, even at relatively low annealing temperatures, for thinner TiO_2_ layers. However, as the amorphous TiO_2_ thickness increases, the lower bulk free energy of anatase becomes dominant, requiring higher thermal energy to induce rutile formation.

We applied the RuO_2_‐top‐interface‐driven strategy to an approximately 1 nm‐thick amorphous ZrO_2_/TiN stack to reduce the EOT. This study also investigated the stabilization of rutile TiO_2_ on amorphous ZrO_2_ surfaces. Figure 2d shows the GIXRD patterns of 25–30 nm‐thick TiO_2_ films grown with a 1.3–5.4 nm‐thick TiO_2_ seed layer on 1 nm ZrO_2_/TiN. The films were annealed at 400°C using the RuO_2_‐top‐interface‐driven strategy, which is compatible with DRAM fabrication. Despite the amorphous nature of the ZrO_2_ layer, an anatase‐to‐rutile transition was observed with decreasing TiO_2_ thickness. Figure 2e shows the scanning transmission electron microscopy (TEM) image of the RuO_2_/TiO_2_ seed layer/ZrO_2_/TiN structure, where the lattice fringes of TiO_2_ were aligned with those of RuO_2_. This coherent alignment was consistently observed at other locations (Figure S5). These results illustrate that the proposed RuO_2_‐top‐interface‐driven strategy can induce the crystallization of rutile TiO_2_ regardless of the underlying layer's type and crystallinity. Furthermore, rutile formation was also verified when the same approach was applied on different underlying layers, such as amorphous SiO_2_ and Al_2_O_3_ layers (Figure S6), further supporting that the crystallization is primarily governed by the RuO_2_ upper layer rather than by the underlying substrate.

The RuO_2_‐top‐interface‐driven approach includes additional steps such as annealing to crystallize TiO_2_ and etching the RuO_2_ upper layer to replace top electrodes, potentially leading to device degradation. Specifically, these post‐deposition procedures may trigger oxidation of the underlying electrodes, such as TiN, which are highly susceptible to oxidative conditions. Therefore, to assess the viability of this RuO_2_‐top‐interface‐driven method, we investigated the possibility of mitigating the oxidation of the underlying TiN electrode during subsequent processes.

**FIGURE 3:**
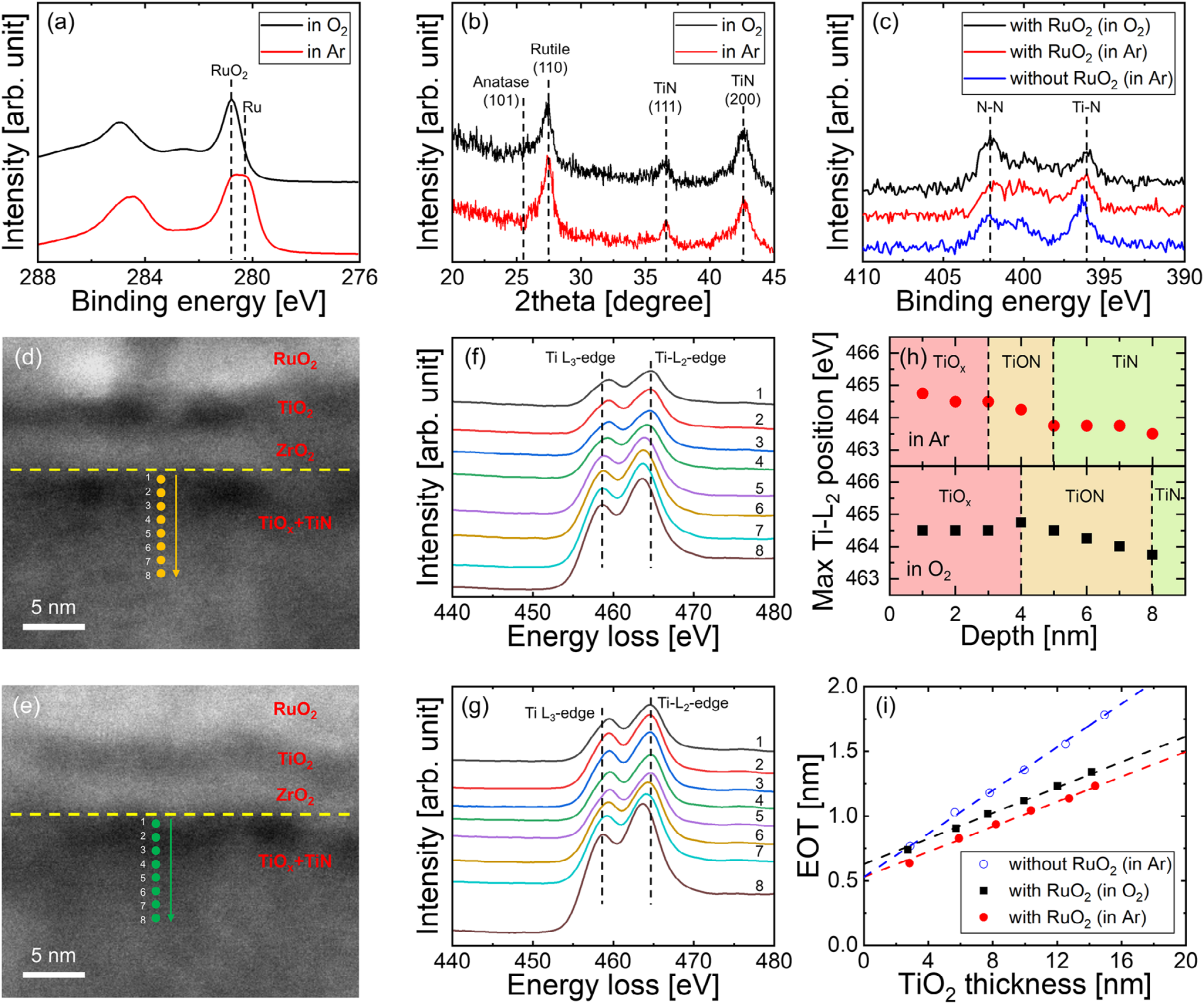
(a) Ru 3d XPS spectra of RuO_2_ films annealed at 400°C in Ar and O_2_. (b) GIXRD patterns of TiO_2_ films grown on 1 nm‐thick amorphous ZrO_2_/TiN stacks after annealing at 400°C in Ar and O_2_. (c) N 1s XPS spectra of TiO_2_/ZrO_2_/TiN stacks after annealing and subsequent RuO_2_ etching, comparing RuO_2_‐assisted crystallization followed by O_2_ annealing, RuO_2_‐assisted crystallization followed by Ar annealing, and Ar annealing without RuO_2_. TEM images of RuO_2_/TiO_2_/ZrO_2_/TiN stacks annealed in (d) Ar and (e) O_2_ atmospheres. Depth‐resolved Ti L_2,3_‐edge EELS spectra collected at 1 nm intervals from the ZrO_2_/TiN interface in the RuO_2_/TiO_2_/ZrO_2_/TiN stacks post‐annealing in (f) Ar and (g) O_2_ atmospheres. (h) Extracted Ti L_3_‐edge EELS peak positions from (f) (upper panel) and (g) (lower panel). (i) Variation in EOT values of TiO_2_/ZrO_2_/TiN stacks with varying TiO_2_ film thickness.

Because the annealing process can impact both the crystallization of TiO_2_ and the oxidation of the underlying TiN, we investigated the influence of the annealing atmosphere. Although no significant changes in the valence states of ZrO_2_ and TiO_2_ beneath the RuO_2_ upper layer were observed under either annealing atmosphere (Figure S7), the phase stability of the RuO_2_ upper layer was notably affected by the annealing atmosphere. The Ru 3d XPS spectra in Figure 3a show that annealing in an O_2_ atmosphere maintains the RuO_2_ phase, whereas annealing in an Ar atmosphere leads to a partial reduction of RuO_2_, resulting in a mixture of Ru and RuO_2_. This behavior is attributed to the limited thermal stability of RuO_2_ at low oxygen pressures [31]. Despite the partial reduction in the Ar atmosphere, the amorphous TiO_2_ films crystallized exclusively into the rutile phase at 400°C under both Ar and O_2_ atmospheres when employing the top‐interface‐driven strategy (Figure 3b). This indicates the effectiveness of the strategy even in the presence of partial RuO_2_ reduction during annealing.

To compare the extent of TiN oxidation under different annealing atmospheres, N 1s XPS spectra were acquired for the TiO_2_/ZrO_2_/TiN stacks post‐annealing and subsequent RuO_2_ etching (Figure 3c). Additionally, the spectrum of a control sample annealed in Ar without a RuO_2_ upper layer is included for reference. A previous study indicated that during the ALD of oxides on a TiN substrate, nitrogen remains unreleased in the oxidized layer, manifesting as a peak around 402 eV in the N 1s XPS spectrum [32]. Hence, the intensity of the peak serves as an indicator of the degree of TiN oxidation. All samples exhibited a peak around 402 eV, signifying the interfacial oxidation of TiN. Notably, the sample annealed in O_2_ displayed a higher peak intensity compared to the other samples, suggesting that annealing in an inert atmosphere is more effective in inhibiting oxidation. Furthermore, the similar peak intensities observed, irrespective of the presence or absence of the RuO_2_ upper layer, imply that the RuO_4_ formed during the partial reduction of RuO_2_ minimally impacts the oxidation of the underlying TiN layer.

To gain deeper insights into the oxidation of TiN under various annealing atmospheres, electron energy‐loss spectroscopy (EELS) analysis was conducted. TEM images in Figure 3d,e depict the RuO_2_/TiO_2_/ZrO_2_/TiN stacks post‐annealing in Ar and O_2_ atmospheres, respectively. The Ti L_2,3_‐edge EELS spectra, acquired at 1 nm intervals from the ZrO_2_/TiN interface downward, illustrate varying depth‐dependent shifts of the Ti L_2,3_‐edges based on the annealing atmosphere (Figure 3f,g). The peak positions, summarized in Figure 3h for a clear comparison, reveal the presence of TiO_x_ and TiON layers at the interface in both samples. Energy‐dispersive X‐ray spectroscopy mapping further validates the interfacial oxidation (Figure S8). Notably, the oxidized layers are significantly thicker in the stack annealed in O_2_. Additionally, nitrogen depletion extends deeper into the TiN layer, with the TiON‐related nitrogen peak appearing at noticeably greater depth in the O_2_‐annealed stack (Figure S9). These findings provide additional evidence that the oxidation of the TiN electrode progresses more extensively under an O_2_ annealing atmosphere.

Figure 3i illustrates the EOT value variations of the TiO_2_/ZrO_2_/TiN stacks concerning the TiO_2_ film thickness. The EOT values were derived from the capacitance measurements (Figure S10). TiO_2_ films, crystallized using the top‐interface‐driven approach, demonstrated a high dielectric constant of approximately 80, regardless of the annealing atmosphere, indicating rutile formation throughout the TiO_2_ layer. Consistently, the refractive index and density also match those of rutile TiO_2_, further supporting homogeneous crystallization of the film (Figure S11). In contrast, films crystallized without the RuO_2_ top layer exhibited a lower dielectric constant of approximately 46, consistent with anatase formation. Notably, the y‐intercept for the stack annealed in Ar is notably lower than that for the stack annealed in O_2_ and matches that of films formed without the RuO_2_ top layer. Consequently, these findings suggest that annealing in an inert atmosphere minimally impacts additional interfacial oxidation during crystallization.

**FIGURE 4:**
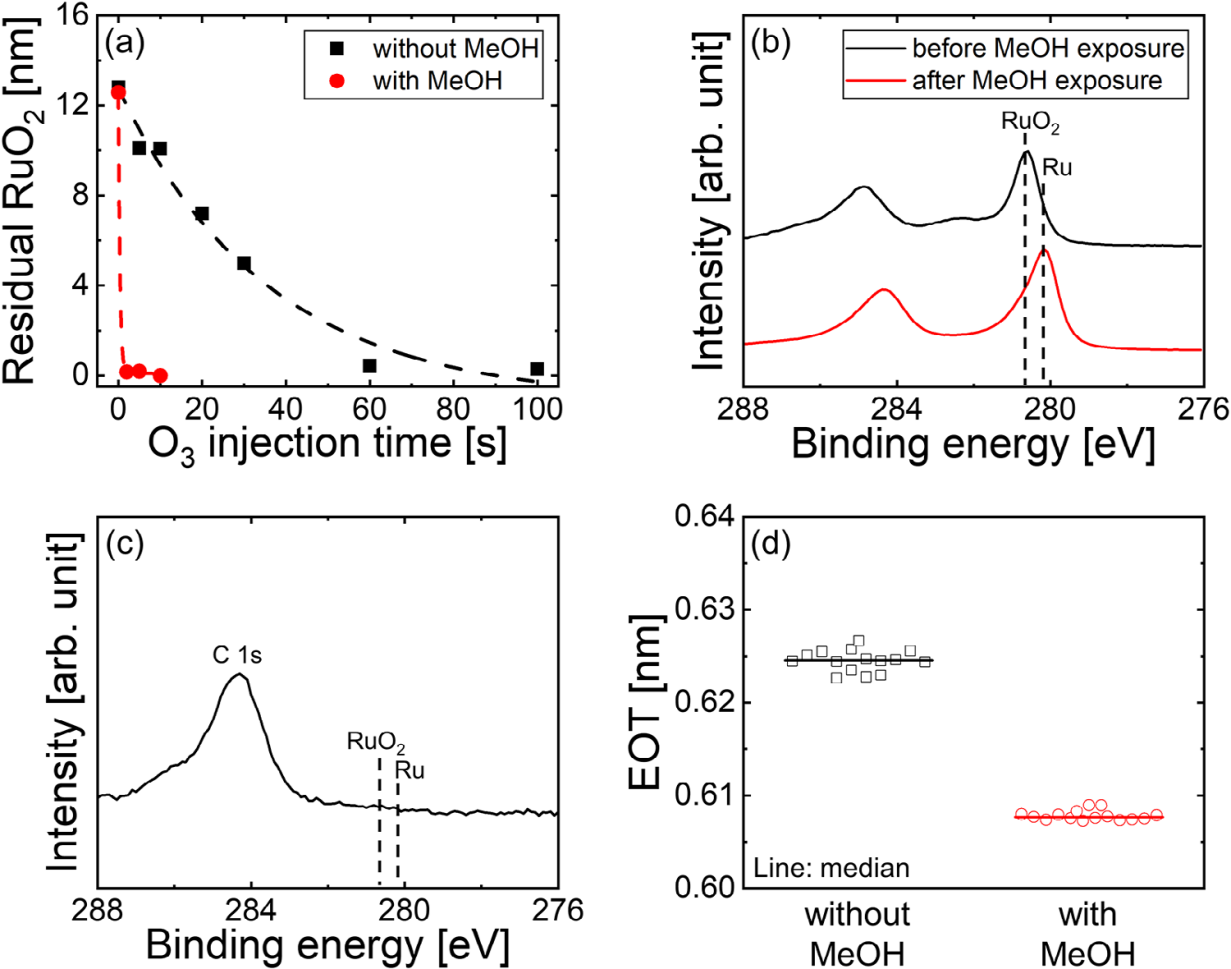
(a) Variation in the remaining thickness of a 12 nm‐thick RuO_2_ layer after etching at 220°C using repeated O_3_ pulses as a function of O_3_ injection time per pulse. (b) Ru 3d XPS spectra of RuO_2_ films before and after 10 s MeOH exposure at 220°C. (c) Ru 3d XPS spectrum of RuO_2_ after 10 etching cycles consisting of MeOH and O_3_ pulses. (d) Comparison of EOT values of 2.3 nm‐TiO_2_/1 nm‐ZrO_2_/TiN stacked capacitors with and without MeOH introduction.

To expand the range of top electrode materials, the RuO_2_ upper layer should be removed without causing degradation to the underlying stack. RuO_2_ can be selectively etched using strong oxidants [33], and thus, O_3_ was used for this purpose. However, the etching rate of RuO_2_ in O_3_ is slow, requiring prolonged exposure that may lead to oxidation of the TiN bottom electrode. Complete etching of an approximately 12 nm‐thick RuO_2_ layer required over 60 s of O_3_ injection per pulse across 10 pulses at 220°C (Figure 4a). Considering the swift etching kinetics of metallic Ru [33] and the thermodynamic preference for O_3_ to react with Ru rather than RuO_2_ (Figure S12), we propose a two‐step etching strategy. In this approach, the RuO_2_ layer is first reduced to Ru by exposure to methanol (MeOH) and then eliminated through O_3_ exposure to minimize oxidation of the underlying TiN layer. A brief 10‐s exposure to MeOH was adequate to completely reduce RuO_2_ to Ru (Figure 4b), indicating the facile reduction of RuO_2_ with MeOH. Based on these findings, a series of 10 cycles involving MeOH and O_3_ pulses enabled the complete removal of the RuO_2_ layer, even with a short 2‐s O_3_ exposure per pulse (Figure 4a), as further confirmed by the absence of Ru in the XPS analysis (Figure 4c). To evaluate the effect of MeOH introduction on dielectric performance, the EOT values of the 2.3 nm‐TiO_2_/1 nm‐ZrO_2_/TiN stacked capacitors were compared with and without MeOH introduction, as shown in Figure 4d). Although the EOT values were comparable, a slight reduction in EOT was noted when MeOH was utilized. This marginal decrease indicates that the proposed etching method is likely to inhibit further oxidation of TiN electrodes.

Thus, this reduction‐assisted etching not only prevents TiN degradation but also enables RuO_2_ to function as a removable crystallization template. Rutile TiO_2_ can be adopted in DRAM capacitor stacks without sacrificing the freedom in selecting the top electrode.

## 3 Conclusion

We presented a top‐interface‐driven crystallization method that stabilizes hard‐to‐form rutile TiO_2_ at temperatures suitable for industry, regardless of the substrate lattice structure. Through the introduction of a RuO_2_ upper layer that matches the structure, we successfully transformed amorphous TiO_2_ into rutile. This approach achieved rutile formation even on amorphous ZrO_2_ and TiN, which had not been possible using traditional bottom‐interface‐driven methods. Additionally, we developed a MeOH‐assisted reduction–etching technique that selectively removes RuO_2_ without oxidizing TiN, facilitating subsequent integration of top electrodes while maintaining dielectric performance.

This study establishes a new paradigm for hard‐to‐form phase control driven by the top interface rather than the substrate, offering a substrate‐agnostic pathway to high‐k rutile formation and a practical route toward DRAM scaling. This methodology could be extended to other polymorphic oxides necessitating metastable stabilization within the ALD thermal windows, thereby creating a versatile framework for designing and integrating metastable materials.

## 4 Experimental

### 4.1 Film Growth

TiO_2_ films were produced in a traveling‐wave‐type chamber (Atomic Classic, CN‐1) using ALD. Amorphous TiO_2_ seed layers were primarily grown at a low temperature of 200°C, while the main TiO_2_ layers were grown at 300°C to facilitate crystallinity identification. Trimethoxy(pentamethylcyclopentadienyl)titanium ((CpMe_5_)Ti(OMe)_3_, provided by SK Trichem Co.) and O_3_ were utilized as the Ti and O sources, respectively. A canister containing (CpMe_5_)Ti(OMe)_3_ was maintained at 43°C, and the (CpMe_5_)Ti(OMe)_3_ molecules were introduced into the chamber using a N_2_ carrier gas at a flow rate of 220 sccm. O_3_ was generated in a discharge‐type O_3_ generator, yielding O_3_ with a concentration of 185 g/Nm^3^ by passing O_2_ gas at a rate of 500 sccm. The injection times for (CpMe_5_)Ti(OMe)_3_ and O_3_ were set at 4 and 3 s, respectively, based on preliminary growth tests confirming self‐saturation behavior. Sputtered TiN and thermally oxidized SiO_2_ were predominantly employed as substrates, onto which an ultrathin ZrO_2_ layer was deposited to serve as a reaction barrier. The RuO_2_ top layer was sputtered onto the amorphous TiO_2_ seed layer at 200°C using DC sputtering, followed by annealing to crystallize the amorphous TiO_2_ into a rutile structure. After crystallization, the RuO_2_ top layer was selectively removed through repeated MeOH‐O_3_ etching cycles at 220°C. Subsequently, an additional 25 nm‐thick TiO_2_ layer was deposited via ALD to aid in phase identification.

### 4.2 Characterization

The films' thicknesses and refractive indices were analyzed using spectroscopic ellipsometry (MG‐5000 NanoView Co.). GIXRD was conducted at an incident angle of 1° to determine the crystal structure of the TiO_2_ films. The crystal structure was further investigated with high‐angle annular dark‐field scanning TEM (Titan 80–300, FEI). Energy‐dispersive X‐ray spectroscopy and EELS (GIF Quantum ER 966, Gatan) were used to map the elemental distribution across the RuO_2_/TiO_2_/ZrO_2_/TiN stack. EELS spectral data were collected within an energy range of 130–640 eV, encompassing the N‐K, Ti‐L, and O‐K edges. The changes in the chemical binding states at the interface were investigated using XPS (Nexsa, ThermoFisher Scientific) with a pass energy of 50 eV using monochromated Al Kα X‐ray. The binding energies of all samples were calibrated using adventitious carbon (C 1s: 284.8 eV). Metal–insulator–metal capacitors were fabricated to estimate the dielectric constants of the TiO_2_ films. RuO_2_ top electrodes with a radius of 200 µm were fabricated via reactive sputtering at room temperature using a lift‐off process. Capacitance measurements were conducted in parallel circuit mode (C_p_‐D) with an Agilent 4294A impedance analyzer at 10 kHz.

## Conflicts of Interest

The authors declare no conflicts of interest.

## Supporting information




**Supporting File**: adma73040‐sup‐0001‐SuppMat.pdf.

## Data Availability

The data that support the findings of this study are available from the corresponding author upon reasonable request
