# Supplementary material for: Rutile Without Substrate Limitations: Top‐Interface‐Driven Crystallization of TiO2
Source: Adv Mater. 2026 Apr 9;38(26):e73040. doi: 10.1002/adma.73040 (PMC13155257; doi:10.1002/adma.73040)
Supplement: Supplementary file 1 — Supporting File: adma73040‐sup‐0001‐SuppMat.pdf. [file ADMA-38-e73040-s001.pdf]

Supporting Information

Rutile Without Substrate Limitations: Top-Interface-Driven Crystallization of TiO_2

*Jihoon Jeon, Jongseo Kim, Seungwan Ye, Seong Keun Kim**

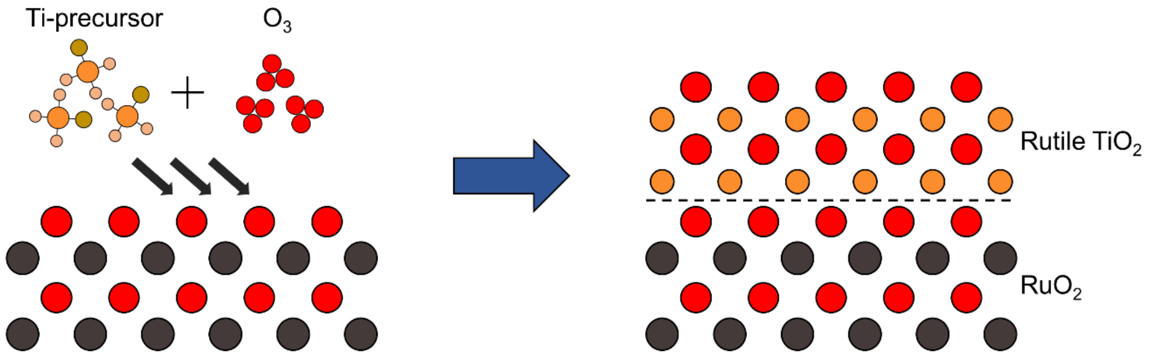

Figure S1. Schematic of rutile formation through lattice matching with the bottom interface.

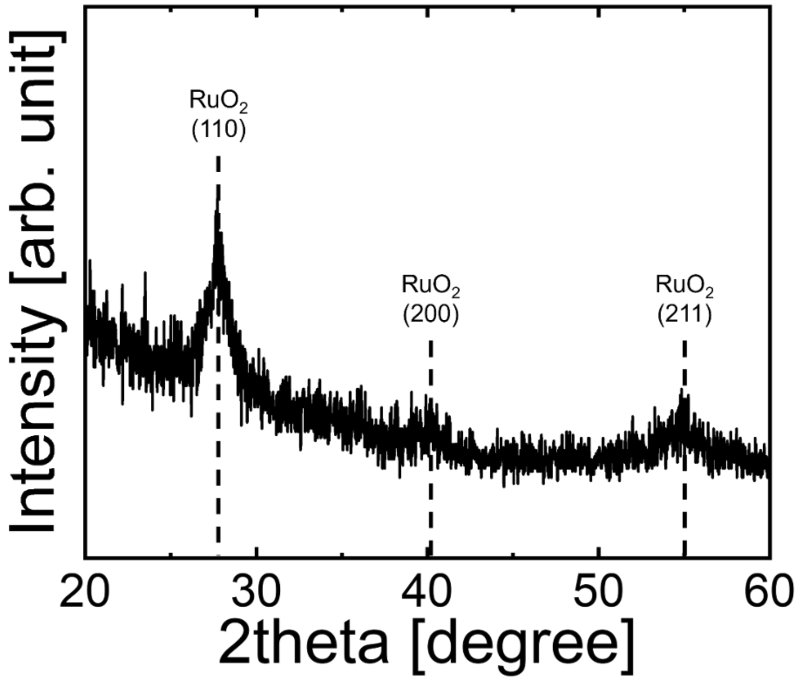

Figure S2. GIXRD pattern of an as-grown 7 nm-thick RuO_2 layer formed on a SiO_2/Si.

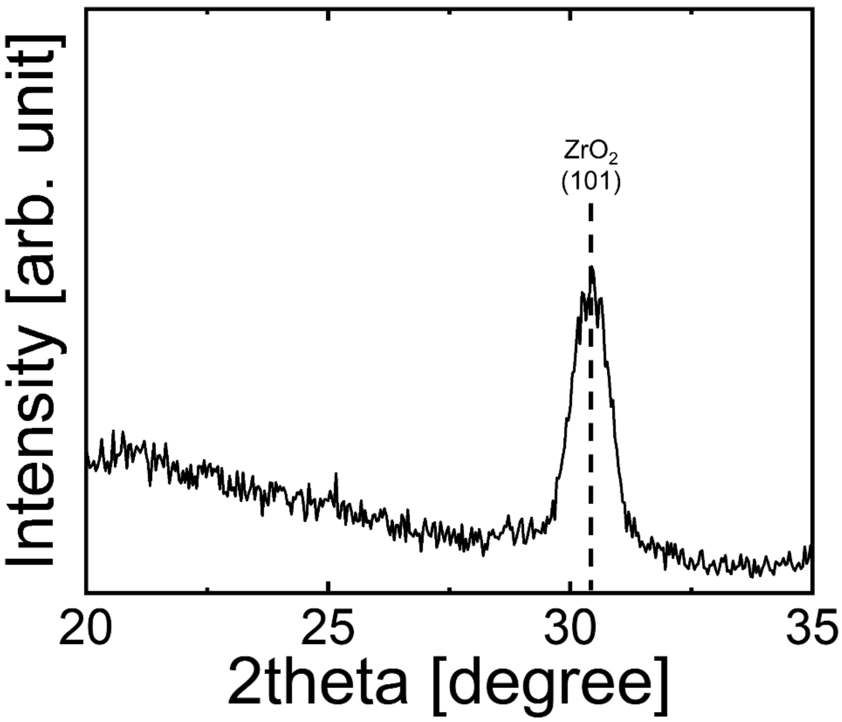

Figure S3. GIXRD pattern of an amorphous 6 nm-thick TiO_2 layer formed on a 10 nm-thick crystalline $ZrO_2/SiO_2/Si$.

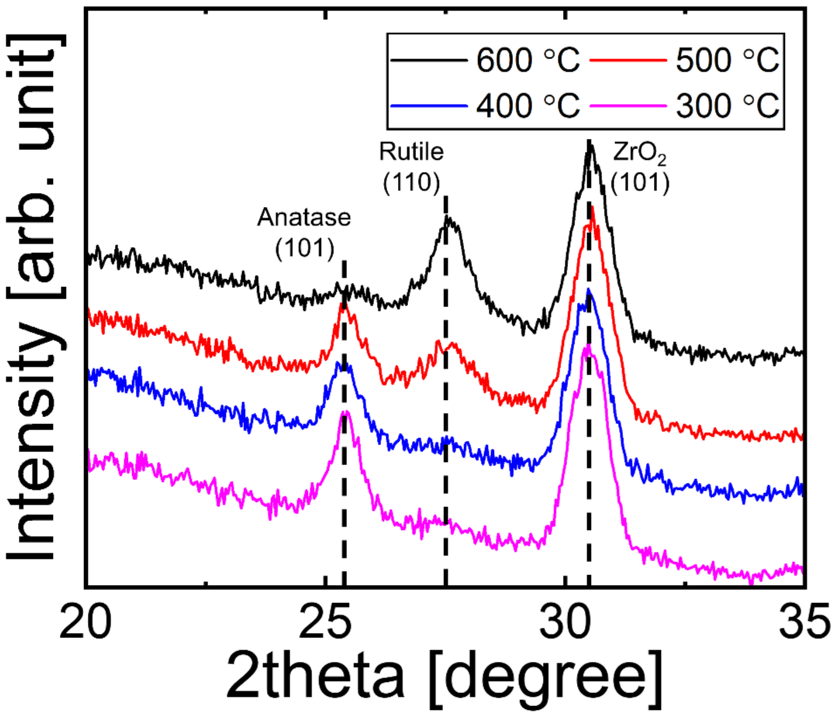

Figure S4. GIXRD patterns of TiO_2 films grown using the top-interface-driven strategy with a 4 nm-thick amorphous TiO_2 seed layer on a 10 nm-thick crystalline $ZrO_2/SiO_2/Si$.

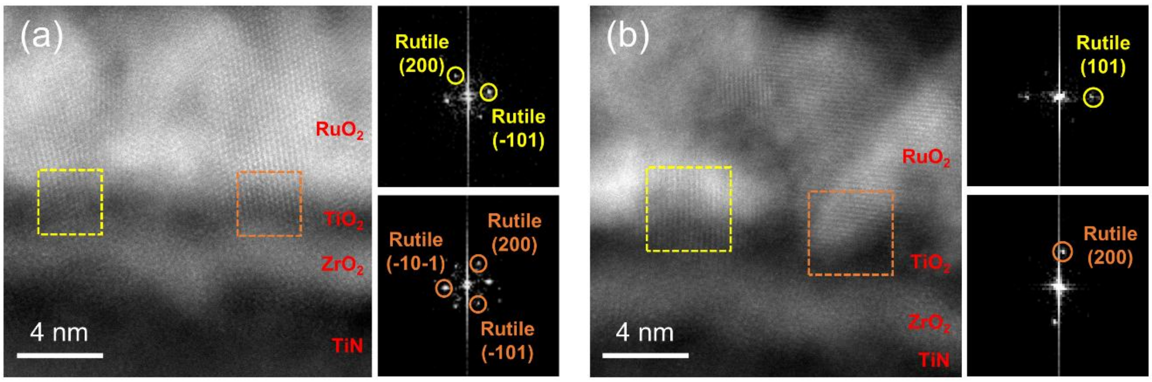

Figure S5. Cross-sectional TEM images of $RuO_2/TiO_2/ZrO_2/TiN$ stacks with amorphous ZrO_2, with insets showing fast Fourier transform images confirming rutile formation.

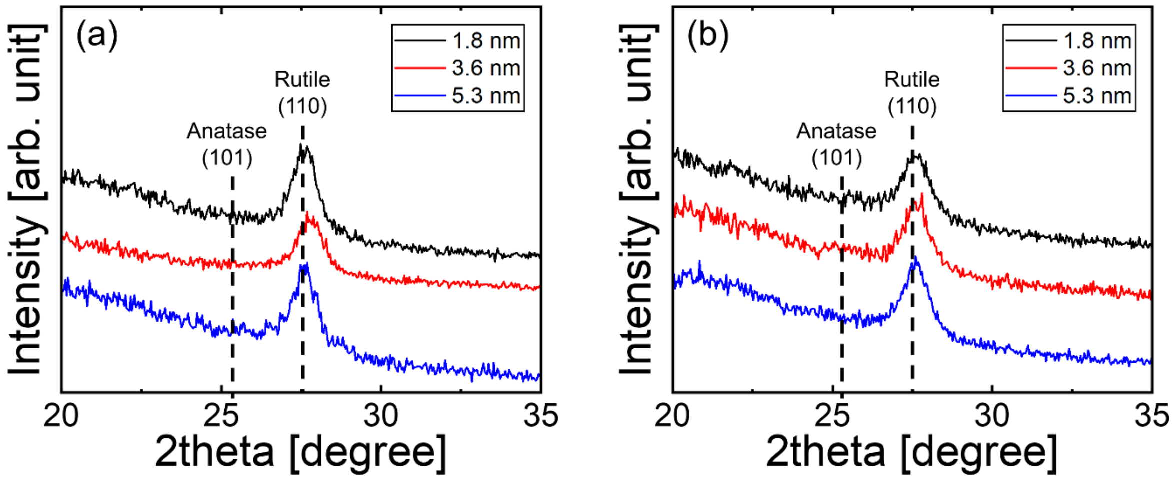

Figure S6. GIXRD patterns of TiO_2 films grown using the top-interface-driven strategy with various amorphous TiO_2 seed layer thicknesses on (a) SiO_2/Si and (b) 2.8 nm-thick Al_2O_3/SiO_2/Si.

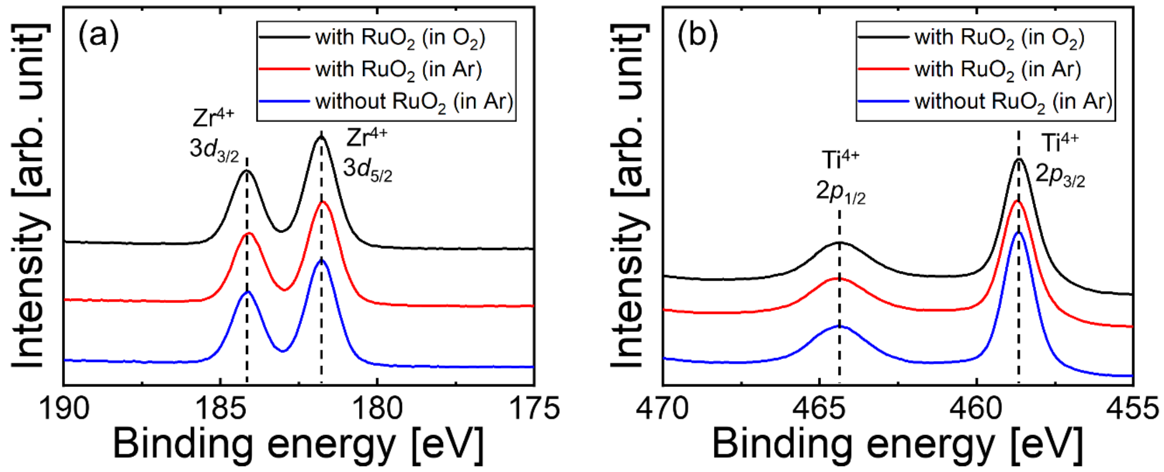

Figure S7. (a) Zr 3d and (b) Ti 2p XPS spectra of TiO_2/ZrO_2/TiN stacks after annealing and RuO_2 etching, comparing RuO_2-assisted crystallization followed by O_2 annealing, RuO_2-assisted crystallization followed by Ar annealing, and Ar annealing without RuO_2.

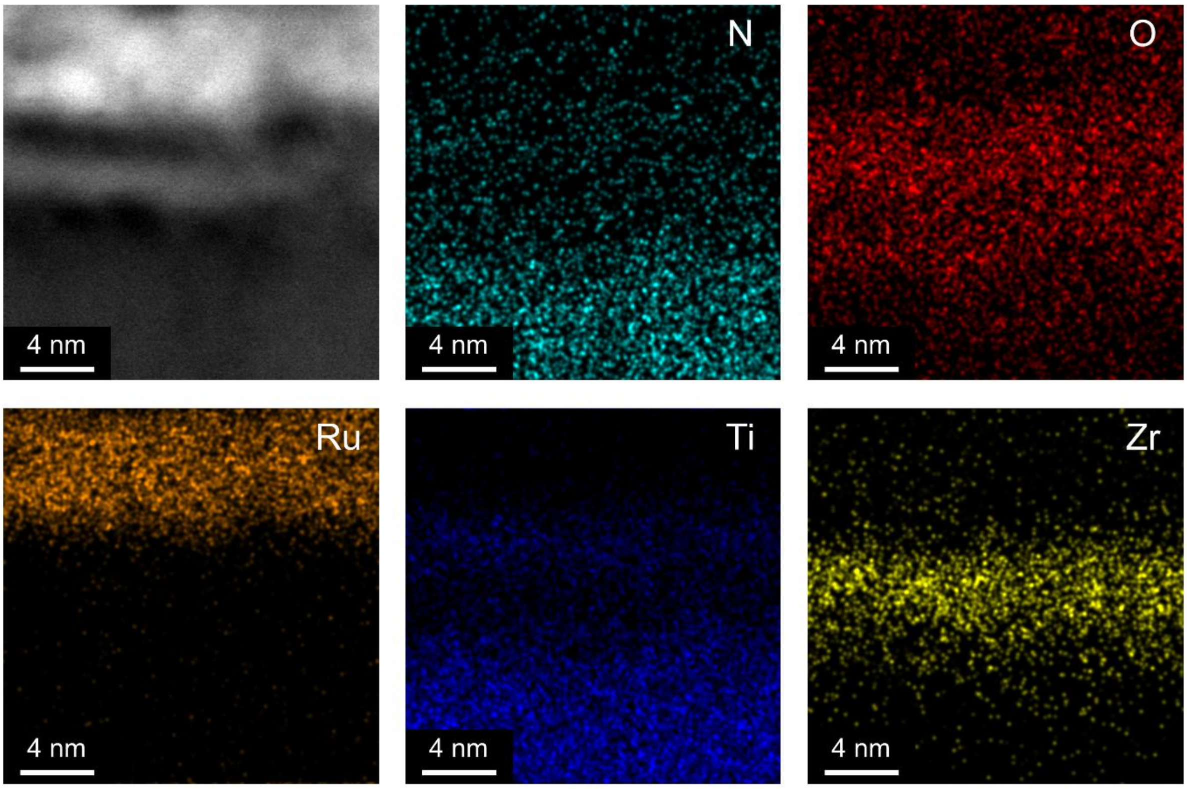

Figure S8. Energy-dispersive X-ray spectroscopy map of a RuO_2/TiO_2/ZrO_2/TiN stack after annealing at 400 °C in Ar.

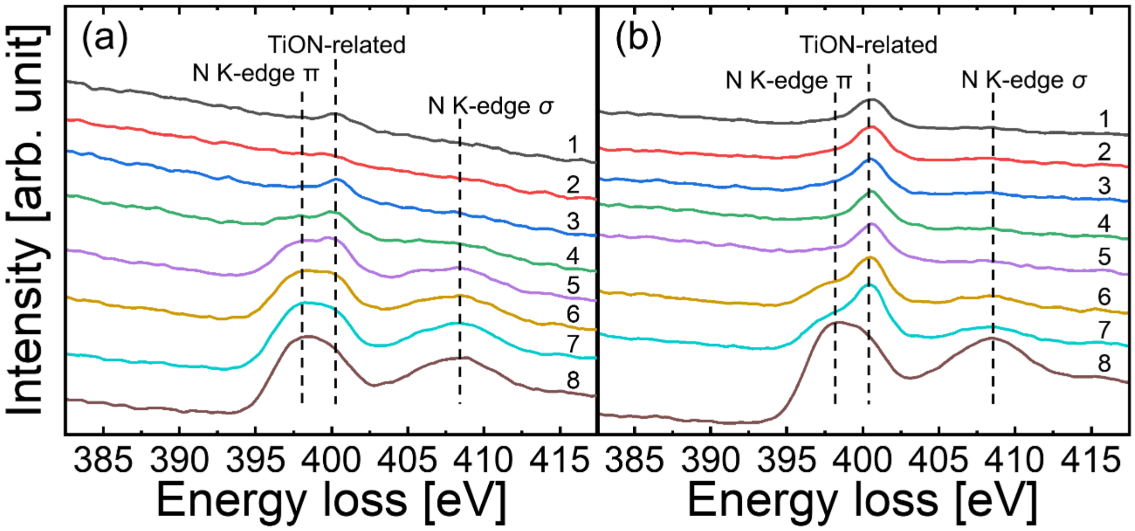

Figure S9. Depth-resolved N K-edge EELS spectra obtained at 1 nm intervals from the ZrO_2/TiN interface in the RuO_2/TiO_2/ZrO_2/TiN stacks post-annealing in (a) Ar and (b) O_2 atmospheres.

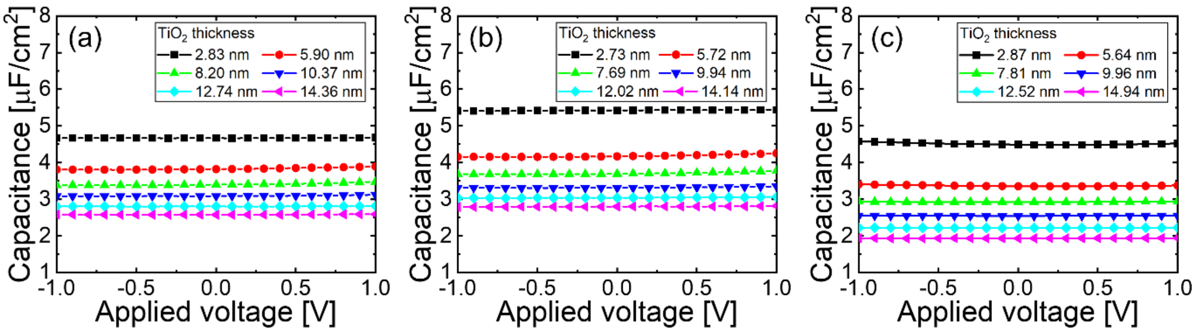

Figure S10. C–V curves of RuO_2/TiO_2/ZrO_2/TiN stacks fabricated using RuO_2-assisted crystallization and annealed in (a) O_2 and (b) Ar, compared with (c) Ar-annealed stacks without RuO_2.

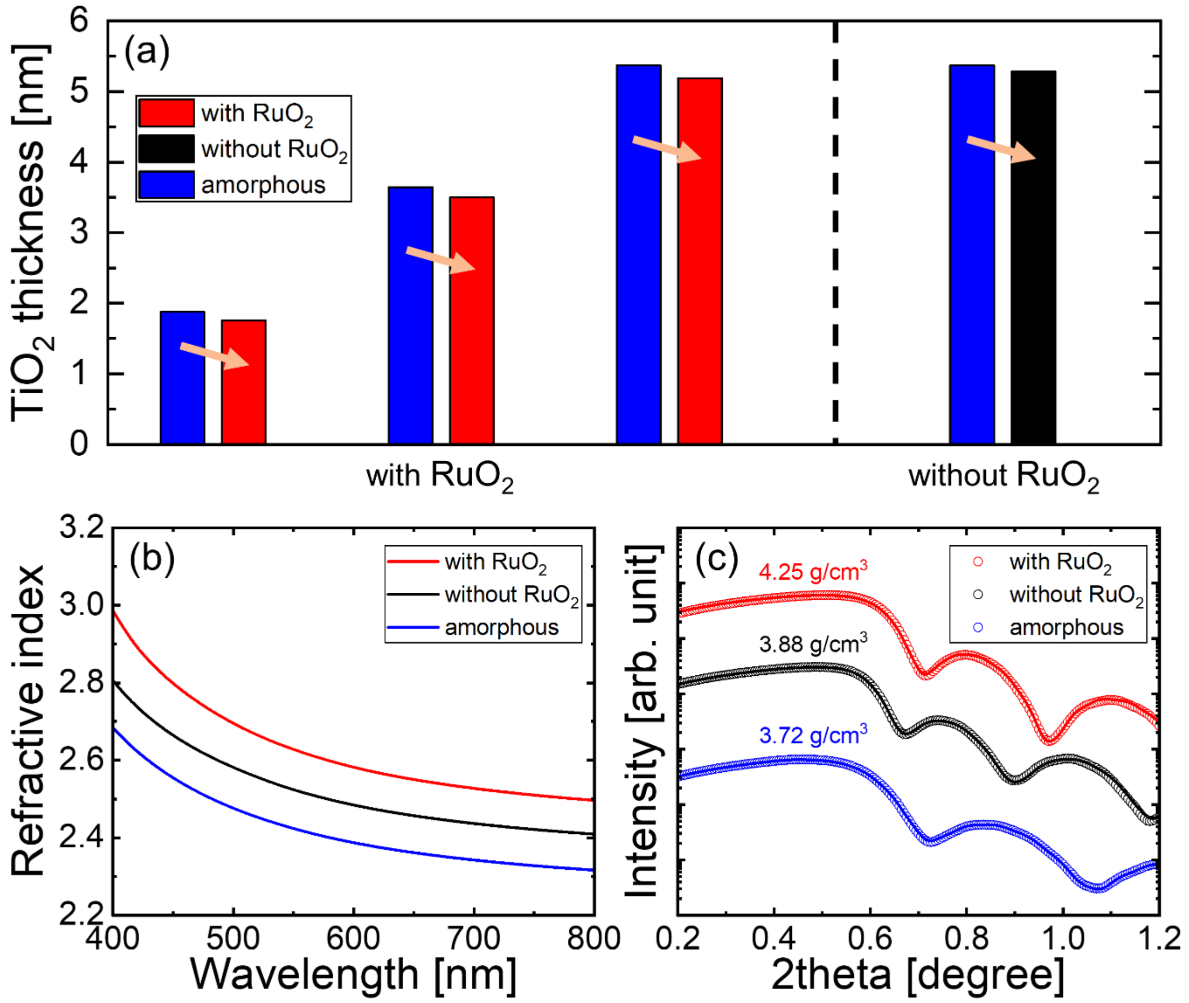

Figure S11. Comparison of (a) thickness, (b) refractive index, and (c) density of the TiO_2 films before and after annealing, for samples processed with and without the RuO_2 upper layer.

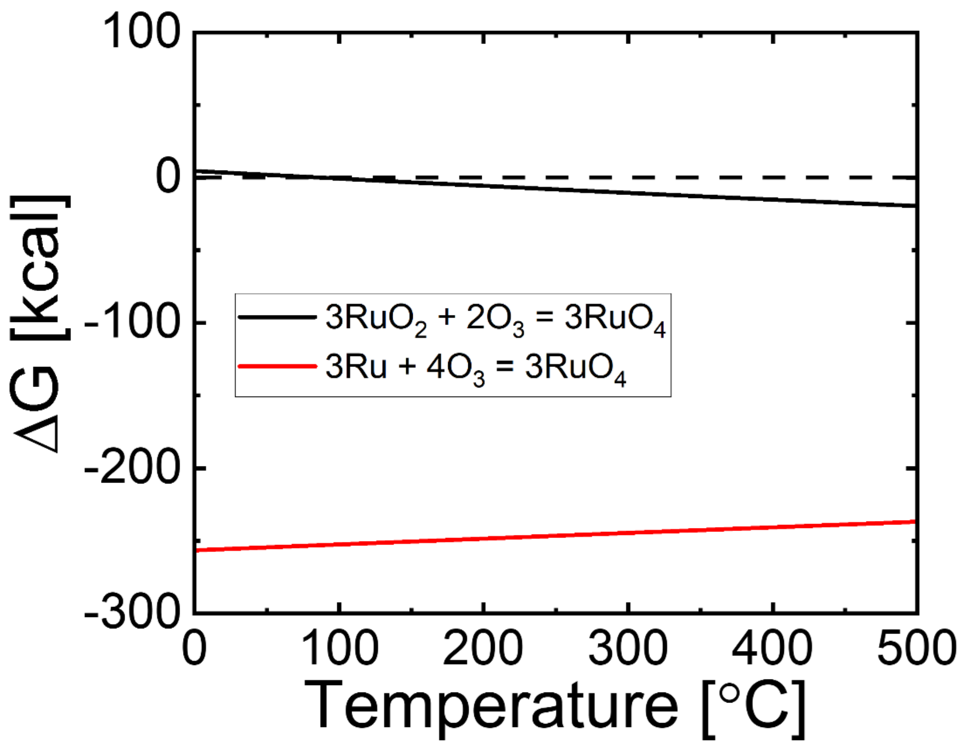

Figure S12. Variation in Gibbs free energy for oxidation of Ru and RuO_2 by O_3 from 0–500 °C.
